# From the United Kingdom to Australia—Adapting a Web-Based Self-management Education Program to Support the Management of Type 2 Diabetes: Tutorial

**DOI:** 10.2196/26339

**Published:** 2022-04-20

**Authors:** Jenny Olson, Michelle Hadjiconstantinou, Carly Luff, Karen Watts, Natasha Watson, Venus Miller, Deborah Schofield, Kamlesh Khunti, Melanie J Davies, Sara Calginari

**Affiliations:** 1 Diabetes WA Subiaco Australia; 2 Department of Kinesiology The Pennsylvania State University University Park, PA United States; 3 College of Medicine The Pennsylvania State University Hershey, PA United States; 4 Diabetes Research Centre University of Leicester Leicester United Kingdom; 5 Catalysis Research and Evaluation Consultancy Perth Australia

**Keywords:** diabetes mellitus, type 2, technology, self-management

## Abstract

Diabetes self-management education and support can improve outcomes in people with diabetes. Providing health interventions via digital modes of delivery can extend the reach of programs delivered through traditional means. The web-based version of the Diabetes Education and Self-Management for Ongoing and Newly Diagnosed (MyDESMOND) is a digital diabetes education and support program for people with type 2 diabetes. The program was originally developed in the United Kingdom and is evidence-based, grounded in behavioral theory, and designed through a rigorous process of intervention mapping. As such, MyDESMOND was considered an ideal candidate for adaptation to the Australian setting. Program content and the digital platform were modified to suit the local context to increase the likelihood that the revised version of MyDESMOND will deliver similar outcomes to the original program. The aim of this paper is to describe the systematic processes undertaken to adapt the digital MyDESMOND diabetes education and support program for people with type 2 diabetes to the Australian setting. The adaptation involved a multidisciplinary group with a diverse range of skills and expertise—a governance structure was established, a skilled project team was appointed, and stakeholder engagement was strategically planned. The adaptation of the program content included modifications to the clinical recommendations, the inclusion of local resources, practical changes, and revisions to optimize readability. A 2-stage independent review of the modified content was enacted. Digital adaptations were informed by relevant standards, local legislative requirements, and considerations of data sovereignty. The digital platform was extensively tested before deployment to the production setting. MyDESMOND is the first evidence-based digital diabetes education and support program for Australians with type 2 diabetes. This paper provides a road map for the adaptation of digital health interventions to new contexts.

## Introduction

### Background

Diabetes is a chronic condition defined by high plasma glucose levels that can lead to an increased risk of serious health complications and premature mortality [1]. The incidence of type 2 diabetes mellitus (T2DM) is increasing worldwide [2]. In Australia, >1.2 million people are estimated to have diabetes [3]. T2DM is the most common form of diabetes [4], with almost 1 million Australian adults diagnosed with this condition [5]. Moreover, it is estimated that there are an additional 500,000 undiagnosed cases of T2DM in Australia and a further 2 million people at high risk of developing the condition in the future [3].

A high proportion of people with T2DM experience long-term complications, including kidney disease, retinopathy, amputations, heart attacks, and stroke, associated with reduced life expectancy [6]. Optimal management of T2DM to reduce the risk of long-term complications requires the adoption and maintenance of self-care behaviors, including healthy eating, physical activity, blood glucose monitoring, medication adherence, and behaviors that reduce the risk of complications (eg, foot checks), in addition to the application of cognitive strategies to facilitate problem solving and healthy coping [7].

Diabetes self-management education and support (DSMES) programs empower people with diabetes to be actively involved in their own self-care by supporting informed decision-making and the adoption and maintenance of self-care strategies and behaviors [8]. DSMES has shown to be effective in reducing glycated hemoglobin A_1c_ (HbA_1c_) levels [9] and decreasing the risk of all-cause mortality [10], as well as being cost-effective compared with usual care [11]. One such program is the Diabetes Education and Self-Management for Ongoing and Newly Diagnosed (DESMOND) program available in the United Kingdom for people with T2DM, which was evaluated in a multicenter randomized controlled trial and was also found to be cost-effective [12,13]. This program was adapted and developed for the Australian population and found to be effective in increasing patient activation (ie, active involvement in self-management of health conditions) among people with T2DM in regional Western Australia (WA) [14]. DESMOND has since been incorporated into the suite of DSMES programs delivered and evaluated nationally throughout Australia [15].

Despite the utility and cost-effectiveness of DSMES programs such as DESMOND, face-to-face programs may not be accessible or suitable for all people with diabetes. In addition, particularly during COVID-19, web-based delivery as an option has been very attractive [16,17]. Common barriers to participation include logistical (eg, time and lack of transport or parking), medical (eg, a person has another health condition that affects their ability to attend), financial (eg, costs associated with getting to a venue), emotional (eg, negative feelings with regard to groups), and cultural barriers (eg, beliefs and language) in addition to a lack of knowledge or no perceived benefit of attending such a program [15]. Moreover, people with diabetes cannot be assumed to be homogeneous in terms of their engagement preferences when seeking participation in a health service [18]. Individualized preferences should be taken into account in the design of support programs and services [19]. The provision of alternative modes of participation is critical when the goal is to maximize participation rates and improve diabetes care.

**Figure 1:**
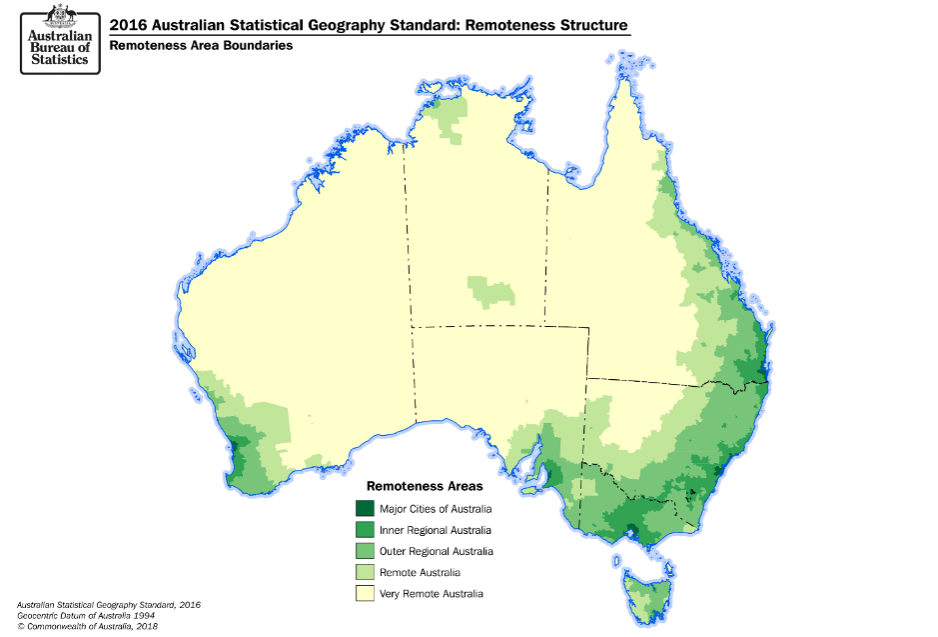
Map of Australia by geographic remoteness.

In Australia in particular, access to programs can be affected by geographic remoteness, which affects the availability of health resources, accessibility, and the financial viability of facilitating programs and services in remote areas. Australia’s population is dispersed over almost 7.7 million km^2^ [20]. To demonstrate the potential impact of geographic remoteness on face-to-face service delivery, a map of Australia by geographic remoteness is presented in Figure 1. Around one-third of the Australian population lives outside major cities [21], with age-standardized ratios of diabetes often higher in regional and remote areas than in major cities [22].

Web-based delivery of DSMES offers an opportunity to provide comprehensive coverage of education and support to otherwise difficult to reach populations. Such programs can mitigate many of the barriers associated with attendance to face-to-face programs, particularly in the Australian context where the population is dispersed over a vast geographical area.

Participation in DSMES via web-based modes of delivery has increased significantly in recent years [23]. Such programs are effective in improving knowledge and glycemic control when compared with usual care [24] and have been associated with improved well-being outcomes in people with T2DM [25]. Hadjiconstantinou et al [26] recently adapted the face-to-face version of DESMOND to a digital platform in accordance with the intervention-mapping approach [27]. The adoption and implementation of this digital program, named MyDESMOND, in the UK setting are currently under evaluation. Early findings suggest that the program is effective in reducing diabetes distress and improving self-efficacy in diabetes self-management [17]. MyDESMOND is an interactive digital program to support people living with T2DM. The digital platform has functionalities including educational material; booster sessions; peer support chat forum; *ask the expert*; integration with commercially available activity trackers; a decision maker (ie, personalized goal setting); and tracking of HbA_1c_, blood pressure, cholesterol, weight, and waist measurements.

### Objective

The aim of this paper is to describe the systematic process undertaken to adapt MyDESMOND to the Australian setting in readiness for consumer pilot-testing. This systematic approach considered the relevant social, cultural, environmental, and policy-level factors of influence to develop the first web-based DSMES program for people with T2DM in Australia. The description of this work provides a road map for others to follow when adapting comprehensive digital self-management programs to local contexts to ensure that such programs meet the needs and priorities of all stakeholders (eg, consumers, service providers, health systems, and governments) and are fit for purpose for implementation in a new setting.

## Methods

### Overview

In December 2018, following a *request-for-quote* process, the Australian Government Department of Health (hereon referred to as *the Department*) commissioned Diabetes WA, National Diabetes Services Scheme (NDSS) Agent for WA, to adapt, pilot, evaluate, and implement MyDESMOND in the Australian setting. The process undertaken to adapt the program to the Australian context is the focus of this paper.

**Figure 2:**
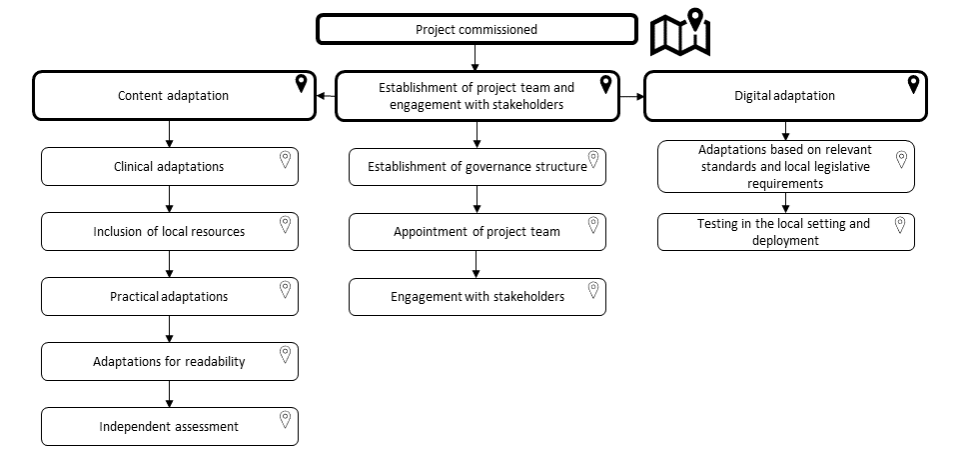
Project roadmap for the adaptation of MyDESMOND.

The adaptation of MyDESMOND to the Australian context involved three key areas of focus: (1) establishment of the project team and carefully planned engagement with stakeholders, (2) content adaptation, and (3) digital adaptation. These areas of focus are summarized in the project road map presented in Figure 2 and described in further detail below.

### Establishment of Project Team and Engagement With Stakeholders

**Figure 3:**
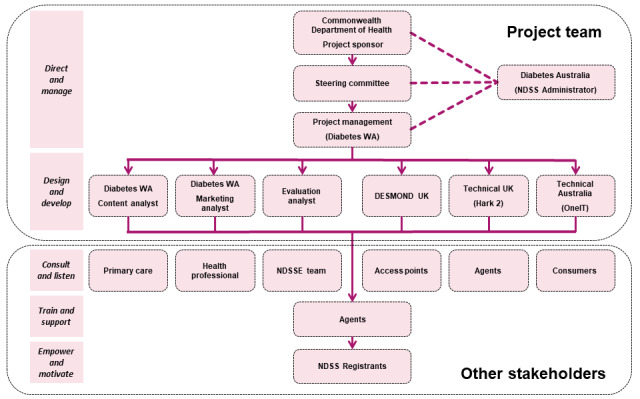
MYDESMOND governance, project management, and stakeholders.

The first stage of the project to adapt MyDESMOND involved the establishment of a governance structure, appointment of the project team, and planning for stakeholder engagement. An organizational chart portraying the roles and responsibilities of the governing bodies, project management team, and key stakeholders is presented in Figure 3 and described in further detail below.

#### Establishment of a Governance Structure

A steering committee of senior stakeholders was established to provide overall governance of the project, including the Chief Executives from Diabetes WA, Diabetes Australia, and Diabetes Tasmania; the General Manager of the NDSS; and the Directors of Diabetes Products, NDSS Enhancements, and Chronic Disease Policy from the Department. The role of the steering committee was to make recommendations in relation to project plans, budgets, and timelines and to broadly monitor progress and risks throughout the life of the project. This included recommendations as to how MyDESMOND (1) integrated with and complemented without duplicating existing NDSS services delivered by Diabetes Australia and Agents of the NDSS across Australia; (2) complied with relevant government policy, the overarching NDSS service agreement, and individual NDSS Agent agreements; (3) met relevant standards for NDSS content, review, and approvals; (4) complied with local data privacy, cyber security, and other internet technology security requirements of the NDSS and government policy; (5) managed risks, defined as any event or condition with the potential to affect achievement of project objectives; and (6) could be delivered in accordance with agreed budgets and time frames.

#### Appointment of the Project Team

Diabetes WA was responsible for the day-to-day operation of the project. A project management team was appointed. The team, led by an experienced project manager, included Credentialled (accredited) Diabetes Educators (CDEs); content, marketing, and evaluation analysts; and a digital systems consultant. The team met weekly and was responsible for all aspects of developing, managing, and monitoring the project work plan and for securing appropriate resources to ensure the successful completion of the project.

#### Engagement With Stakeholders

A working group comprising representatives of the state and territory Agents of the NDSS, Diabetes Australia, and the Department was also established. The purpose of the group was to foster communication, collaboration, and input into planning for the adaptation, eventual pilot-testing, and implementation of MyDESMOND at the operational level. The group provided advice and support to the project management team to ensure that the program complemented and integrated with existing education and support services and materials provided through the NDSS, met appropriate standards for content, and included appropriate processes for approval and review.

A plan to guide stakeholder engagement and communication was developed and approved by the Agent working group and steering committee to ensure optimal stakeholder awareness, engagement, motivation, and satisfaction. The development of the plan involved four key processes: (1) identification of internal and external stakeholders likely to be affected by project processes or outcomes; (2) analysis of the level of interest, influence, and involvement in the project; (3) consideration of how to best manage stakeholder expectations during the project to maximize support and minimize the potential for conflict; and (4) planning to regularly update and review stakeholder expectations and building strategies to maintain stakeholder engagement and support throughout the life of the project. The plan included a matrix identifying key project stakeholders in addition to the type of information to be communicated, appropriate channels for communication, and the planned frequency and timing of communication activities. The matrix can be viewed in Multimedia Appendix 1.

### Content Adaptation

#### Overview

The content of MyDESMOND was adapted to ensure that the program was (1) culturally and contextually appropriate for the Australian setting, (2) consistent with the content of the face-to-face version of DESMOND already being delivered in Australia, and (3) aligned with Australian clinical guidelines for the management of T2DM. The content was adapted by a CDE who was also accredited to facilitate the face-to-face adaptation of DESMOND in Australia and by an experienced content analyst. The process initially involved a detailed review of the UK version of MyDESMOND to identify program elements requiring adaptation. The program content was also compared with the Australian *DESMOND Newly Diagnosed and Foundation: Educator Manual and Curriculum 2015* [28] previously adapted for face-to-face delivery of DESMOND in the Australian context. As a result of this work, adaptations were made to program text, quizzes, videos, fact sheets, and the decision maker and health tracker (ie, biomedical) parameters. This process resulted in (1) clinical adaptations, (2) inclusion of local resources, (3) practical adaptations, and (4) adaptations for readability, followed by (5) a 2-step process of independent assessment.

#### Clinical Adaptation

The clinical guidelines for the management of T2DM cited throughout MyDESMOND were amended in accordance with Australian recommendations and guidelines. Targeted HbA_1c_ levels were amended from 6.5% to 7% (48-53 mmol/mol) [29]. Blood glucose targets were amended from UK standards (ie, 4-7 before meals and <8.5 two hours after) to 6 to 8 mmol/L fasting and 8 to 10 mmol/L after meals [29]. References to medications were updated to reflect those available in Australia [29,30]. Smoking was included in the list of known risk factors for the development of T2DM [29]. Content describing nutritional information was also adapted. For example, information about the fat content of packaged food included on food labels was amended to reflect local standards [31]. Australian guidelines for physical activity and sedentary behavior were referenced [32] in addition to Exercise and Sport Science Australia’s position statement on exercise prescription for patients with T2DM and prediabetes [33].

#### Inclusion of Local Resources

Links and references to UK-focused consumer resources were replaced with links and references to Australian resources. For example, references to services and resources provided through the National Health Service (NHS) in the United Kingdom were replaced with references and links to the services and resources provided through the NDSS in Australia. These included references to the NDSS website landing page and information about blood glucose monitoring; management of hypoglycemia; sick days; medications; food labels; physical activity; sexual health and diabetes; the diabetes annual cycle of care; and advice on driving, travel, healthy cooking, and eating out [34]. Other local resources included information from the Heart Foundation [35], Nutrition Australia [36], and the Australian Government dietary guidelines [31] and physical activity guidelines [32].

#### Practical Adaptation

A variety of additional practical adaptations were also made to MyDESMOND. These included the conversion of all units of measurement to be consistent with Australian standards. For example, calories were changed to kilojoules, and ounces were changed to grams. Spelling and grammar were also edited to reflect locally accepted conventions (eg, *programme* was changed to *program*). Branding throughout the program was amended from that of the NHS and the Leicester Diabetes Centre to that of the Australian Government Department of Health and the NDSS. References accrediting the Leicester Diabetes Centre for the original program design were retained. Embedded videos were refilmed to reflect the changes to program content and local language accents and included the stories of Australian people living with T2DM. Images were adapted to suit the Australian context. To ensure consistent formatting throughout the program, the images were edited by the Leicester Diabetes Centre. Processes for registration were also updated to be contextually relevant. This included data to be collected in relation to the participants’ postcode information (ie, Australian postcodes), gender (ie, male, female, and X—*other*), ethnicity (ie, including response options for Aboriginal and Torres Strait Islanders), and registrant identification (ie, NDSS registration number rather than NHS number).

#### Ensuring Readability

Given Australia’s multicultural diversity, including migrants of non–English-speaking background, it was important to ensure that MyDESMOND was appropriate for people with lower levels of English literacy. Each learning session and booster session throughout the program was reviewed against the NDSS readability checklist [37]. The purpose of the checklist is to ensure that the content of NDSS materials is suitable for people living with diabetes with low levels of English proficiency and health literacy. The checklist includes items describing appropriate use of language (eg, *Choose words that are familiar and culturally appropriate for your readers*), sentence structure (eg, *Use one idea per sentence*), type of information to be included (eg, *Tell your readers how to get more help or information*), design features (eg, *Choose a colour scheme that is not distracting*), and pictures (eg, *use pictures, logos or photographs to add meaning to the text*).

The readability of MyDESMOND was also assessed using the Flesch–Kincaid Scale [38], a scale that has commonly been applied to assess the readability of health information materials [39]. The score was calculated using the proofing feature in Word for Microsoft 365 [40]. The targeted reading level was equivalent to grade 7-9 (approximate age 13-15 years) as specified in the NDSS readability standards [37]. Specific content assessed at a level higher than this was revised, ensuring that the language, length, and complexity of the sentences were appropriate for the targeted level of readability. Furthermore, to enhance the accessibility of MyDESMOND, we presented content in a variety of formats, including text, videos, video transcripts, and simple pictures.

#### Independent Assessment

Upon completion of the amendments to the program content, MyDESMOND was subjected to a 2-stage process of independent assessment to confirm that the content was appropriate for the Australian context and complied with all relevant standards and guidelines. First, a review was conducted by 2 external CDEs (ie, these CDEs were not affiliated with Diabetes WA or otherwise involved in the MyDESMOND project). The program was then sent to the Medical Education and Scientific Advisory Council for review. The Medical Education and Scientific Advisory Council is a body maintained by the Australian Diabetes Educators Association and the Australian Diabetes Society and provides strategic advice to the NDSS on medical, educational, and scientific matters. The results of these 2 stages of review confirmed that the content of MyDESMOND was consistent with relevant standards and guidelines and, therefore, likely to be suitable for Australian consumers.

### Digital Adaptation

#### Overview

**Figure 4:**
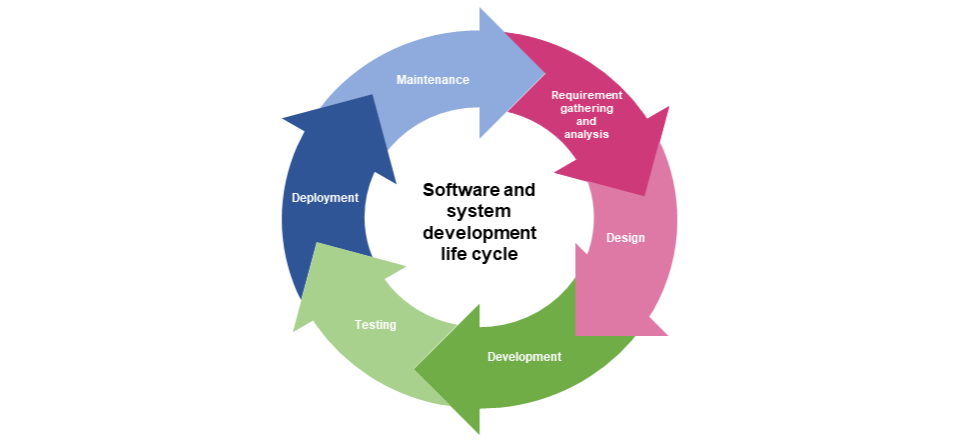
Software development life cycle.

The digital specifications of MyDESMOND were modified to suit the Australian setting. This part of the project was overseen by a member of the project management team who was an experienced project systems consultant. Coding and development work outside of the content management system was undertaken by the UK-based developers of the original MyDESMOND platform. The modifications were guided by a software and system development life cycle approach. Such approaches offer a stage-based conceptualization of the life of a system or software from inception to maintenance [41]. The format of life cycle models and the terms applied to describe the stages of the life cycle vary between models depending on the context and intended application [41]. As shown in Figure 4, stages may include requirement gathering and analysis, design, development, testing, deployment, and maintenance.

The requirement gathering and analysis phase of a life cycle typically involves identification of stakeholder needs and building understanding of how users will interact with the system and how the system should function. Given that MyDESMOND had been developed and offered to people with T2DM in the United Kingdom, this phase of the project involved ensuring that the program met local standards and legislative requirements for information and data management. The design phase of the life cycle usually involves the determination of a high-level system design, whereas the development phase involves the configuration of the infrastructure as well as database and system coding. In the case of MyDESMOND, this work had been undertaken by a UK-based web developer when the program was originally developed. The next phase of the life cycle involved testing to verify that the system worked as expected under conditions simulating *real life*. Deployment occurs when system functionality is verified and involves the release of the software or system to end users. When a system is deployed and users start to interact with it, issues or defects may be identified, at which point the life cycle continues to evolve.

#### Adaptations Based on Relevant Standards and Local Legislative Requirements

MyDESMOND was adapted to meet relevant standards and local legislative requirements for best-practice information and data management. Relevant standards and legislation informing local adaptations included (1) the National Institute of Standards and Technology [42] information system security plan template; (2) the Australian Government protective security policy framework [43], including policies related to sensitive and classified information, access to information, safeguarding information from cyber threats, and robust information and communication technology systems; (3) the Australian *Privacy Act 1988* [44] and Privacy Principles [45], including standards, rights, and obligations around the collection, use, and disclosure of personal information, the organization’s governance and accountability, the integrity and collection of personal information, and the rights of individuals to access their personal information; and (4) infrastructure and software security controls aligned with the Australian Government information security manual [43] for the requisite information classification.

The review of relevant standards and local legislation resulted in changes to the program privacy policy and terms and conditions and the addition of a feature to enable users to opt in or out to receive future direct marketing campaigns. More specifically, the amendments included (1) strengthening passphrase (password) management practices for user accounts, including the adoption of protocols for the frequency of required changes to passphrases and limitations on the frequency of user-instigated daily password changes, the reuse of passphrases, and the use of sequential passphrases; (2) implementation of multifactor authentication; (3) recording of relevant information for logged events, including a description of the event, the date and time it occurred, and the users, processes, and equipment involved; and (4) implementation of the Secure Hash Algorithm 2 family of Secure Hash Algorithms [46] for enhanced data security.

To ensure data sovereignty, whereby MyDESMOND data were stored and accessible only from within Australia [47], the application was installed on Australian servers in accordance with the Secure Shell Protocol [48]. The Secure Shell Protocol ensures secure remote logging and network services over insecure networks through processes of authentication and encryption.

#### Testing

The Australian MyDESMOND platform was tested to validate that the program functioned as specified and, thus, could be considered *production-ready*. A test plan outlining the strategies for testing and execution and the processes for test management was developed. The plan was reviewed by key stakeholders before its execution to foster commitment to resourcing and time frames.

Testing focused on the functionality of the platform (ie, the application functioned as intended), capacity for backup and recovery (ie, verifying that the system had appropriate methods for data replication and that data could be effectively restored if required), and penetration (ie, identification of vulnerabilities in the infrastructure and software that might be exploited to compromise the confidentiality, integrity, or availability of the system). Additional nonfunctional testing typically conducted at this stage of the development life cycle (eg, availability, reliability, and performance) was not required as the MyDESMOND Australia installation had been configured to replicate the UK environment, which had been operating nationally in the United Kingdom since 2018 with >15,000 users (well in excess of anticipated user numbers for the Australian pilot).

The project team developed test cases for each function and feature of MyDESMOND to identify inputs and expected outputs, allow for remediation of issues where the actual output varied from anticipated outputs, and identify opportunities to improve user experience. The cases were manually executed by professional test engineers using a range of common web browsers (eg, Google Chrome, Safari, Internet Explorer, and Firefox) and devices (eg, desktop and laptop computers and iOS and Android phones and tablets). Project team members participated in testing (eg, project managers and coordinators, CDEs, marketing coordinators, and evaluation coordinators). Testing was repeated when system shortfalls were identified and rectified. In total, 736 test cases were executed on web browsers, and 566 were executed on devices. More than 100 issues were reported and categorized according to severity (eg, low, medium, and high). No issues were categorized as *high*. Examples of issues included duplication of words, poor rendering of display, inaccurate or unhelpful error messages, incorrect navigation, inappropriate or missing verification and validation, and problems connecting with third-party services. Upon completion of testing, MyDESMOND was profiled as *low-risk* and determined as functionally fit for purpose in the Australian setting. The disaster recovery plan was successfully tested, with the system fully recovered and restored. Specialist internet technology security consultants conducted penetration testing to simulate a malicious user both with and without credentials. This test demonstrated that MyDESMOND was not susceptible to cyberattacks and would not expose users to unacceptable risk. Upon completion of functional, disaster recovery, and penetration testing, MyDESMOND was recommended for deployment to the production environment in preparation for consumer pilot-testing.

## Discussion

### Principal Findings

The steps outlined in this paper facilitated the cultural adaptation of MyDESMOND, the first evidence-based web-based DSMES program for Australian people living with T2DM. By documenting the systematic approach, including the appointment of a multidisciplinary project management team, active stakeholder engagement, and the specific processes undertaken to adapt program content and the digital platform, this paper provides a road map for others to follow when adapting digital health programs for delivery in new contexts.

Adaptation of existing evidence-based health programs to new settings has the potential to introduce services into new environments in a cost-effective and timely manner with reasonable prospects of successful outcomes [49]. However, health interventions interact with features of the context in which they are embedded; it cannot be assumed that an intervention that is effective in one setting will be effective in another setting [50]. Adaptations to suit the characteristics of the new setting can enhance the likelihood that the intervention will achieve similar outcomes to those realized in the original setting [50]. The adaptation of MyDESMOND optimized the program for delivery in the Australian setting. The approach undertaken—including the selection of an existing suitable and effective program; review of original program materials; identification of localized objectives, core components, and mismatches between the original program and new context; and adaptation of original materials—was consistent with established frameworks for the adaptation of traditional health programs for use in new contexts [49].

The incorporation of stakeholder input into the design of behavioral interventions to improve public health is critical, as is the development of strategies for optimal and sustained stakeholder engagement [51]. The engagement of a diverse group of stakeholders in the contextual adaptation of MyDESMOND facilitated input and *buy-in* from funders, service providers, health professionals, and others who stood to be affected by the implementation of the program in the short and long term. Inclusion of these parties from project instigation to piloting, evaluation, and implementation improves the likelihood of ongoing *buy in* and support from those who have influence over program delivery, thereby enhancing the potential sustainability of the program in the future.

### Strengths and Limitations

A strength of this work is the adoption of an existing evidence-based program grounded in behavioral theory and designed using a systematic intervention-mapping approach [26]. It is recommended that health intervention design be informed by theoretical frameworks [27], and evidence shows that theory-based interventions are effective in improving HbA_1c_ levels, self-efficacy, diabetes knowledge, and self-care behaviors in people with T2DM [52]. Thus, when choosing an appropriate digital DSMES program to support Australians with T2DM, MyDESMOND was an ideal candidate. MyDESMOND Australia is evidence-based, underpinned by behavioral theory, and optimized for delivery in the Australian setting, thereby increasing the likelihood that it will be effective in improving outcomes in Australians with T2DM.

Robust security and privacy protections for the rights of consumers are key to ensure that digital health interventions are effective, safe, and trusted by users; a proactive approach that considers potential and emerging threats, relevant standards and legislative requirements, and evolving ethical issues is essential [53]. Accordingly, the identification of relevant standards and laws for digital information, combined with extensive localized testing of the MyDESMOND platform, is a strength of this work. Moreover, the appropriateness of the adaptations to program content and the digital platform included processes for external review and system testing, respectively. The incorporation of these processes provided checks and balances to confirm that MyDESMOND was likely to be contextually appropriate and suitable for use among Australians with T2DM.

We followed a systematic approach to adapt MyDESMOND to the Australian setting. However, there are limitations that need to be acknowledged. When working with project stakeholders, it is critical to collaboratively establish and agree on detailed timelines, systematic work plans, and goal-setting processes with continual revision of standards set and group norms [27]. Despite a priori planning of strategies for optimal stakeholder engagement, the high level of project governance by senior stakeholders from a range of external organizations with competing agendas led to delays in the establishment of the initial MyDESMOND project plan. In addition, although the 2-stage review process of program content provided some assurance of the cultural appropriateness of the adaptations, the time taken by external assessors to review and provide feedback was underestimated, leading to further project delays. However, these delays did not affect the quality or overall timeline of the project.

Delays were also experienced in adapting the MyDESMOND digital platform owing to a lack of system administrator training or hand over and lack of architectural diagrams and administrator guides or description of access levels. This resulted in a process of trial and error as the local project team learned how to navigate the system. To mitigate this unforeseen issue, we engaged a content editor experienced in technology and digital communications to ensure that our team developed and followed robust system user guides. Involving the UK developers of the original MyDESMOND platform in the project from the outset also provided an additional source of knowledge and expertise to overcome this issue. Consequently, we recommend involving original program developers as stakeholders when adapting digital programs to new settings, where possible.

Although the adaptation of MyDESMOND Australia involved a collaborative approach and extensive stakeholder engagement, program users (ie, NDSS registrants) were not directly involved in the adaptation at this stage. However, follow-up pilot-testing involves an initial exploratory phase to seek preliminary feedback about program acceptability from users before advancing to more comprehensive phases of pilot-testing.

### Key Recommendations When Adapting Digital Programs

We provide a list of recommendations to consider for future adaptation work in digital self-management programs (Textbox 1). These recommendations can be applied to other long-term health conditions and cultural settings.

**Key recommendations to consider for the adaptation of future digital self-management programs.**

**Key recommendations for the adaptation of digital self-management programs**

- Involve individuals with multidisciplinary skills and expertise in the project management team. This may include those experienced in project management, program or clinical content, and digital systems and processes in addition to the original program developers.
- Involve stakeholders across multiple levels of influence. This might include funding or commissioning bodies, senior executives and operational-level employees of service-providing organizations, health professionals and clinicians, original program developers, and others who may have an interest in or be affected by program implementation.
- When working with independent advisors and bodies, it is critical to establish workable time frames and deadlines and seek agreement to those time frames by all concerned.
- Create a clear plan for how stakeholder engagement will be sustained throughout the life of the project. Consider the priorities and agendas of each of the stakeholders.
- Use information and resources from a range of credible local sources to inform content adaptations. These may include government guidelines and recommendations as well as information and resources from peak industry bodies, advisory councils, and professional societies.
- When adapting an existing digital program to a new setting, it is important to ensure that the relevant team members have been provided with adequate system administration training and that appropriate technical user guides and resources are supplied.
- Incorporate a process for external or independent review of program content to ensure that it is culturally appropriate and relevant to the target population.
- Adopt a systematic approach to digital adaptations to ensure optimal performance in the local environment. Consider all relevant standards, local legislative requirements, and matters surrounding data sovereignty.
- Conduct rigorous testing of the digital program to ensure that it performs as intended in the local setting.

### Future Directions

Further work is needed to ensure the feasibility of delivering MyDESMOND in the Australian setting in addition to establishing the acceptability and potential effectiveness of the program among Australian people with T2DM. Consumer pilot-testing was undertaken upon completion of the adaptation project described in this paper and will be reported in detail at a later date. Briefly, the aims of the 2-phase pilot were to (1) determine if MyDESMOND provided a useful, engaging, and relevant learning experience for people in Australia living with T2DM; (2) obtain consumer feedback on the functional and technical aspects of the program; (3) determine the potential effectiveness of MyDESMOND in increasing diabetes empowerment and reducing diabetes-related distress among Australians with T2DM; and (4) further assess consumer satisfaction with MyDESMOND after further adaptations were made based on the findings of phase 1 of pilot-testing. Should consumer pilot-testing indicate that MyDESMOND is acceptable, feasible, and likely to be effective, the program will be recommended for inclusion in the suite of programs and services provided to people with T2DM throughout Australia via the NDSS.

### Conclusions

This paper provides a road map for the adaptation of digital health programs for delivery in new contexts, including recommendations for engaging the project management team and stakeholders and processes for adapting content and the digital platform. We believe that the systematic approach that we adopted to adapt MyDESMOND to the Australian setting would be applicable to other development studies that aim to contextually adapt an existing digital program.

## Appendix

Multimedia Appendix 1 Stakeholder Engagement Matrix.

## Abbreviations

CDE: Credentialled Diabetes Educator
DESMOND: Diabetes Education and Self-Management for Ongoing and Newly Diagnosed
DSMES: diabetes self-management education and support
HbA_1c_: glycated hemoglobin A1c
MyDESMOND: web-based Diabetes Education and Self-Management for Ongoing and Newly Diagnosed
NDSS: National Diabetes Services Scheme
NHS: National Health Service
T2DM: type 2 diabetes mellitus
WA: Western Australia
