# Supplementary material for: From the United Kingdom to Australia—Adapting a Web-Based Self-management Education Program to Support the Management of Type 2 Diabetes: Tutorial
Source: J Med Internet Res. 2022 Apr 20;24(4):e26339. doi: 10.2196/26339 (PMC9069279; doi:10.2196/26339)
Supplement: Multimedia Appendix 1 [file jmir_v24i4e26339_app1.docx]

## Appendix A

### Stakeholder Engagement Matrix

The matrix identifies key stakeholders who are required to be engaged through the development of the MyDESMOND project. It also outlines, at a high level, the information needs, appropriate channels, and frequency/timing of communication activities.

| Stakeholder / Audience | Impact | Influence | Key Content/ Information Requirements | Medium/Channel | Responsibility | Timing/ Frequency |
| --- | --- | --- | --- | --- | --- | --- |
| Australian Government Department of Health, Chronic Disease Policy Section (Project Sponsor) | High | High | - Strategic Direction - Deliverables and Milestones - Progress Reports - Approvals | - Project Steering Committee Meetings - Project Reports | Project Steering Committee | Monthly Project Steering Committee meetings (teleconference)  3-month progress and evaluation reports |
| Project Steering Committee (Diabetes Australia Project Owner) | High | High | - Terms of Reference - Agenda - Project Plans - Budgets - Timelines - Reports - Risks & Issues - Deliverables & Milestones - Minutes and Action Items | - Project Steering Committee meetings - Project Reports | Diabetes Australia | Monthly meetings (teleconference)  3-month progress and evaluation reports |
| Project Team  (Diabetes WA) | High | High | - Activity Plans - Agenda - Risks & Issues - Deliverables and Milestones - Status Report - Minutes and Action Items - Roles and Responsibilities | - Face-to-Face team meetings - Email - Sharepoint team site | - Project Manager | - Weekly Team Meetings - Daily Collaboration via email |
| Diabetes Australia NDSS Agent Working Group | High | High | - Terms of Reference - Agenda - Activity Plans - Timelines - Deliverables & Milestones - Risks & Issues - Minutes and Action Items - Best practice training guides - Communications and Marketing toolkit | - NDSS Agent Working Group Meetings - Emails - Newsletters | - General Manager NDSS | - Monthly Meetings (teleconference or face to face) |
| NDSSE Team | High | High | - Activity Plans for IT Integration - IT Risks & Issues - Deliverables & Milestones | - Project Steering Committee meetings - NDSS Agent Working Group meetings | - Diabetes Australia - Project Manager | - Monthly |
| NDSS Agents | High | High | - Training Guides, FAQ’s - Communications toolkit - Key Messages | - NDSS Agent Working Group Meetings | - NDSS Agent Working Group Representative | - Monthly |
| NDSS Registrants | High | High | - FAQs - User guide - Program features and benefits - Technical support | - Existing NDSS consumer channels - Consumer Reference Groups - Online Surveys - Telephone Support - Social Media | - NDSS Agents NDSS Helpline - Project Team - National Evaluation Team | - Ongoing |
| Leicester Diabetes Centre (UK) | Medium | Low | - Licencing Requirements - Adaptation Requirements - Lessons Learned | - Skype - Videoconferencing | - DWA General Manager – Health Services | - Monthly |
| IT Developers – UK and Australia | High | Medium | - Requirements - Deliverables | - Email - Videoconferencing | - Project Manager | - Weekly (for initial project phases) |
| NDSS Helpline | Medium | Medium | - Training Guides, FAQ’s - Key Messages | - Email - Teleconference | - Diabetes Australia - Project Manager | - As required |
| National Evaluation Team | High | Medium | - Activity Plan - Deliverables and Milestones | - Email - Face to face meetings | - Project Manager | - Weekly |
| Outer regional, remote and very remote Registrants | Medium | Low | - NDSS Registration and benefits - Program features and benefits - Referral Pathways | - Information for local clinical care teams such as GP’s or remote area nurses - Mobile screening buses - Internet access support | - NDSS Agents | - Prior to national roll out |
| Health Professionals including peak organisations such as Australian Diabetes Educators Association ADEA, ADS, RACGP | Medium | Low | - Program features and benefits - Referral pathways | - Existing Health Professional channels e.g. GPs, RACGP guidelines, GP Management Plans, journals - University channels e.g. Diabetes education courses, medical students and company representatives who have contact with Health Professionals that can refer the program | - NDSS Agents Diabetes Australia | - Prior to national roll out |
| Primary Health Networks (PHNs), State Health Bodies and Local Government | Medium | Low | - Program features and benefits - Referral pathways | - Existing channels e.g. Diabetes WA Primary Care Advisory Committee (GP’s, Practice Manager, Practice Nurse, PHN and Rural Health West representation) | - NDSS Agents | - Prior to national roll out |
